# Sensor-Based Assessment of Quadriceps EMG-Amplitude-to-Torque Ratios at Different Knee Angles: An Exploratory Cross-Sectional Study

**DOI:** 10.3390/s26144568

**Published:** 2026-07-18

**Authors:** Zhaoxiang Zhang, Małgorzata Tomicka, Xiaodan Guo, Mingmao Li, Marcin Krawczynski, Piotr Aschenbrenner, Adam Kawczyński

**Affiliations:** 1 Doctoral School, Gdansk University of Physical Education and Sport, Kazimierza Górskiego Street 1, 80-336 Gdansk, Poland; guoxiaodan38@gmail.com (X.G.); mingmao.li@awf.gda.pl (M.L.); 2 Department of Biomechanics and Sport Engineering, Gdansk University of Physical Education and Sport, Kazimierza Górskiego Street 1, 80-336 Gdansk, Poland; malgorzata.tomicka@awf.gda.pl (M.T.); marcin.krawczynski@awf.gda.pl (M.K.); piotr.aschenbrenner@awf.gda.pl (P.A.); 3 Faculty of Medicine, Wrocław University of Science and Technology, 50-370 Wrocław, Poland; adam.kawczynski@pwr.edu.pl

**Keywords:** surface electromyography, wireless EMG, dynamometry, sensor-based assessment, Biodex, OptoJump, activation-to-torque ratio, knee extension, countermovement jump, neuromuscular assessment

## Abstract

Surface electromyography (sEMG) normalized to mechanical output can describe electrical activation amplitude relative to torque, but such ratios should not be interpreted automatically as neuromuscular efficiency. This exploratory cross-sectional study compared quadriceps activation-to-torque ratios at 45° and 75° knee flexion and examined hypothesis-generating associations with countermovement jump (CMJ) outcomes. Seventeen participants performed two brief unilateral maximal isometric knee-extension trials at each angle. Wireless sEMG from the vastus lateralis (VL), vastus medialis (VM), and rectus femoris (RF) and Biodex torque were acquired separately and manually matched by participant, knee angle, and test set. Ratios were calculated as Noraxon-exported mean EMG amplitude divided by Biodex average peak torque. Ratios were higher at 45° than at 75° for VL (mean difference = 0.74 μV/(N·m), BCa 95% CI: 0.34–1.34; Holm-adjusted *p* = 0.020), VM (0.52, 0.09–0.72; *p* = 0.009), and RF (0.69, 0.22–1.32; *p* = 0.028). Exploratory Spearman correlations with CMJ outcomes ranged from −0.38 to 0.32 and were nonsignificant after false-discovery-rate correction. The findings indicate protocol-specific, angle-dependent differences, whereas CMJ associations remain inconclusive and require larger, reliability-focused studies with synchronized acquisition and fully documented signal processing.

## 1. Introduction

Isometric knee-extension torque is influenced by knee joint angle because changes in knee flexion alter quadriceps muscle length, tendon configuration, patellar mechanics, moment arm, and the operating region of the muscle–tendon unit on the force–length curve [1,2,3,4]. These mechanical factors mean that the same level of neural activation may not produce the same torque at different knee angles. Angle-specific assessment is therefore important when interpreting maximal voluntary isometric contractions, especially when the aim is to understand how the quadriceps produce force under different muscle-length conditions.

Surface electromyography (sEMG) provides complementary information about muscle activation during force production, but its interpretation depends strongly on sensor placement, signal conditioning, amplitude normalization, and transparent reporting [5,6,7,8]. Raw or normalized sEMG amplitude alone cannot indicate how much mechanical output was produced, and recent consensus recommendations emphasize that EMG should not be treated as a direct force measure without considering muscle state and mechanical context [9]. Two individuals may show similar sEMG amplitudes but different torque values, or similar torque values with different activation demands. For this reason, the activation-to-torque ratio, calculated as EMG amplitude divided by torque, can be used as a descriptive index of the electrical activation demand associated with producing a given amount of mechanical output.

The interpretation of this ratio requires caution. In some contexts, a lower activation-to-torque ratio may indicate that less electrical activation is required for a given torque, whereas a higher ratio may suggest greater neural drive, less favorable mechanics, reduced force transmission, or greater measurement variability. The related electromechanical-efficiency literature also shows that ratios combining electrical and mechanical signals are methodologically heterogeneous and should be interpreted in relation to the specific task and calculation approach [9,10]. Therefore, in the present study, we avoid treating the ratio as a direct measure of “better neuromuscular efficiency”. Instead, we use the more conservative term activation-to-torque ratio and interpret higher values as greater activation demand per unit torque.

The quadriceps muscle group also has muscle-specific anatomical and functional characteristics. The vastus lateralis (VL) and vastus medialis (VM) are monoarticular knee extensors, whereas the rectus femoris (RF) crosses both the hip and knee joints; therefore, RF behavior may be affected by combined hip–knee configuration as well as knee angle alone [3]. Consequently, VL, VM, and RF may not respond identically to changes in knee angle. Comparing these muscles at 45° and 75° knee flexion may therefore provide useful information about angle-specific activation strategies during maximal isometric knee extension.

Explosive tasks such as the countermovement jump (CMJ) require rapid coordination of muscle activation, force transmission, inter-joint sequencing, stretch–shortening-cycle behavior, and tendon contribution. Rate of force development and early-phase neural drive are important contributors to explosive performance [11,12,13,14,15]. However, CMJ performance is a whole-body task, whereas isometric knee-extension testing isolates one joint action. The CMJ was therefore included as a broad external functional comparison rather than as a mechanically equivalent test, and any associations with the single-joint activation-to-torque ratios were expected to be modest.

Previous studies have reported relationships among maximal strength, RFD, EMG-derived activation, and jump performance, but the magnitude and direction of these relationships vary across populations, measurement systems, and normalization approaches [16,17,18]. In particular, it remains uncertain whether angle-specific quadriceps activation demand during isometric knee extension is meaningfully related to CMJ variables such as jump height, relative peak power, maximum velocity, or RFD.

**Figure 1:**
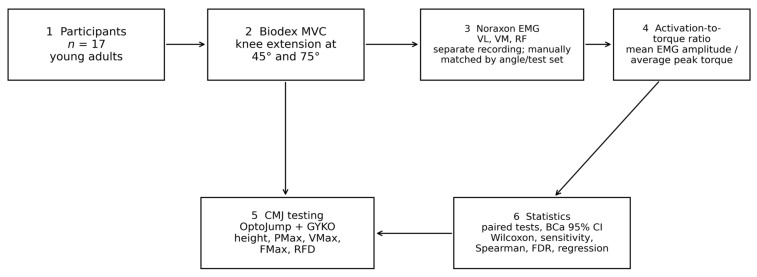
Study workflow. After a standardized 10 min cycle-ergometer warm-up and task familiarization, participants completed Biodex maximal voluntary isometric knee-extension contractions at 45° and 75° knee flexion while surface EMG was recorded separately from VL, VM, and RF. The Biodex and Noraxon records were manually matched by participant, knee angle, and test set; no electronic synchronization or sample-level temporal alignment was available. Activation-to-torque ratios were calculated as Noraxon-exported mean EMG amplitude divided by Biodex average peak torque, and exploratory associations with CMJ outcomes from the best valid hands-on-hips trial were then examined.

Accordingly, this exploratory cross-sectional study had two aims. First, we compared VL, VM, and RF activation-to-torque ratios between 45° and 75° knee flexion during maximal isometric knee extension. Second, we examined whether these protocol-specific, single-joint ratios showed preliminary associations with selected CMJ outcomes. The CMJ analyses were included to explore possible correspondence with a widely used whole-body performance task; they were not intended to test a direct physiological mechanism, establish predictive validity, or validate the ratio. Because of the limited mechanistic correspondence between the tests, the small sample, and the number of associations examined, the CMJ analyses were treated strictly as hypothesis-generating. The study workflow is summarized in Figure 1.

## 2. Materials and Methods

### 2.1. Participants

Seventeen participants completed the study. Participants reported no current lower-limb injury or neuromuscular disorder and no structured high-intensity lower-limb resistance training during the preceding six months. Written informed consent was obtained before participation. Body composition, including body mass and fat-free mass, was assessed using multi-frequency bioelectrical impedance analysis (InBody 720, Biospace Co., Ltd., Seoul, Republic of Korea). The InBody-derived body mass was used for body-mass-normalized outputs where applicable, including torque/body mass and relative CMJ variables.

### 2.2. Isometric Peak Torque

Isometric knee-extension torque was assessed using a Biodex System 4 Pro dynamometer (Biodex Medical Systems, Shirley, NY, USA). Participants were tested in a seated position with the trunk and pelvis stabilized against the backrest. The tested thigh and lower leg were secured using straps to minimize accessory movement, and participants were instructed to hold the stabilization handles during each contraction. The dynamometer axis was visually aligned with the lateral femoral epicondyle, and the lever arm was attached to the distal lower leg proximal to the malleoli. Body mass measured by InBody was entered into the Biodex testing workflow when body-mass-normalized torque outputs were required. Although the Biodex report template displayed an extension/flexion pattern, only isometric knee-extension contractions were performed and analyzed. Gravity correction was not applied in the Biodex export; therefore, the torque values were analyzed as standardized Biodex-reported torque outputs obtained under the same laboratory protocol for all participants.

Unilateral maximal voluntary isometric knee-extension contractions were performed at 45° and 75° knee flexion, with 0° defined as full knee extension. Before the recorded neuromuscular testing, participants completed a 10 min low-intensity cycle-ergometer warm-up. The manufacturer and model of the ergometer were not retained in the archived protocol; the device was used only for warm-up and not for outcome measurement. Participants then completed task familiarization and received verbal instruction. The protocol consisted of two sets, one at each angle. At each angle, participants completed two 3 s extension contractions separated by a 10 s relaxation period. During each attempt, standardized verbal encouragement was provided, including instructions to push as fast and hard as possible and then maintain maximal effort for the 3 s contraction. These instructions were used to standardize effort across repetitions while the Biodex and EMG measurements were manually coordinated during the corresponding test conditions. Average peak torque (N·m) from the two valid extension repetitions at each angle was used as the denominator for activation-to-torque ratio calculation; when a repetition was invalid, only valid repetitions were retained.

### 2.3. Surface Electromyography

Surface EMG was recorded during the MVC testing using a TeleMyo 2400 DTS wireless EMG system (Noraxon USA Inc., Scottsdale, AZ, USA) operated through MyoResearch XP Master Edition (software version unavailable in the archived records). The archived Noraxon acquisition setting showed an EMG sampling frequency of 1000 Hz. The Biodex System 4 Pro and the Noraxon EMG system were operated as separate acquisition systems. A Biodex System 3/4 interface and torque, angular velocity, and angle channels were configured in MyoResearch XP; however, because of a technical connection problem, valid Biodex torque, velocity, and angle outcomes were not recorded in the Noraxon files. Biodex torque was therefore obtained from the separate Biodex reports, whereas EMG amplitude was obtained from the Noraxon export. The records were manually matched by participant, knee angle, and corresponding test set; no hardware/software synchronization, common time base, or sample-level temporal alignment was available. Before electrode placement, the skin was shaved when necessary, gently abraded, and cleaned with alcohol to reduce impedance and improve signal quality. EMG was recorded from the vastus lateralis (VL), vastus medialis (VM), and rectus femoris (RF). Electrodes were placed according to standardized anatomical landmarks and general sEMG placement recommendations [5,7]. Archived screenshots from the MyoResearch XP Master Edition configuration are available as Supplementary Figure S1. Panel A shows the EMG trace view and a manually marked MVC analysis period. Panel B documents the configured Biodex interface and channel settings only and should not be interpreted as evidence that valid Biodex outcomes were recorded in MyoResearch XP. The third EMG channel is labeled 15 in the trace view and 13 in the configuration view; this discrepancy is retained transparently and is not used to infer complete record-to-record channel consistency. The screenshots also show display/feedback smoothing controls (100 ms in the trace view and 1000 ms in the feedback configuration); because the archive does not establish that these controls represent the signal-processing filter applied to the exported amplitude, they were not interpreted as evidence of a specific filter type or order.

For VL, electrodes were positioned at the midpoint between the greater trochanter and the lateral border of the patella. For VM, electrodes were positioned approximately 3–4 cm medial to the patella over the distal–medial quadriceps muscle belly. For RF, electrodes were positioned midway between the anterior superior iliac spine (ASIS) and the superior border of the patella. Electrode placement was kept consistent across participants as close as possible, and cables/sensors were secured to reduce movement artifact during maximal contractions [5,7].

EMG amplitude values were obtained from the archived Noraxon Standard EMG Analysis export. The export contained EMG amplitude time-series data for the selected channels, marked valid analysis periods, and the summary output ‘Averaged Mean Amplitude of All Periods’ in μV. For each muscle and knee angle, the activation-to-torque ratio was calculated by dividing the Noraxon-exported mean EMG amplitude obtained from the marked valid MVC periods by the Biodex average peak torque calculated from the two valid MVC trials in the corresponding manually matched test condition. Because the archived records did not fully document all internal preprocessing settings, such as exact filter type, filter order, rectification procedure, and amplitude-processing algorithm, the EMG variable is reported conservatively as Noraxon-exported mean amplitude rather than as a fully reconstructable raw-signal RMS or MAV value. A concise summary of the preserved and unavailable acquisition, manual data-matching, and signal-processing information is provided in Supplementary Table S2.

### 2.4. Countermovement Jump

Countermovement jump performance was assessed using the OptoJump Next system (version 1.7.0; Microgate, Bolzano, Italy) combined with a GYKO Pro inertial device (Microgate, Bolzano, Italy). The GYKO sensor was used according to the manufacturer’s testing workflow and synchronized with the OptoJump system. The system was used to evaluate lower-limb performance variables relevant to the CMJ, including jump height, relative peak power (PMax, W/kg), maximum velocity (VMax, m/s), maximum force (FMax, N/kg), and CMJ-derived rate of force development (RFD, N/kg/s). The reported RFD variable refers to the CMJ-derived value exported by the OptoJump/GYKO system. Although OptoJump-based systems can also provide temporal variables such as contact and flight time in other protocols, only the CMJ variables included in the present analysis were reported.

After the standardized warm-up and task familiarization, participants performed three maximal CMJ trials from an upright standing position with the feet placed approximately hip- and shoulder-width apart and the hands maintained on the hips throughout the movement to reduce arm-swing contribution. Before each trial, participants were instructed to stand still, keep the trunk as upright as possible, perform a rapid downward countermovement to a self-selected knee-flexion depth of approximately 90° when possible, and then immediately jump vertically as high as possible. They were also instructed to take off and land with both feet on the OptoJump measurement area and to avoid tucking the knees during flight. Verbal encouragement was provided for each attempt. When a trial showed an obvious technical error, such as loss of hand position, visible imbalance, or an incomplete landing, the trial was repeated. The best valid trial, defined by the highest jump height, was retained for statistical analysis, and the corresponding OptoJump/GYKO-derived variables were used in the correlation and regression analyses. OptoJump has demonstrated strong concurrent validity and excellent test–retest reliability for vertical jump height assessment, with previously reported validity ICCs of 0.997–0.998 and test–retest ICCs of 0.982–0.989 [19]. OptoJump Next has also been reported to provide reliable temporal measurements such as contact and flight time, although system settings can influence measurement precision and should be considered when interpreting temporal variables [20]. The GYKO inertial sensor has also been examined for concurrent validity in vertical jump assessment [21].

### 2.5. Statistical Analysis

Descriptive statistics are reported as mean ± SD. The main angle comparison was defined a priori as the paired within-participant difference between 45° and 75° knee flexion for each quadriceps muscle. Mean paired differences were calculated as 45° minus 75°, so positive values indicate higher activation-to-torque ratios at 45°. Paired comparisons between 45° and 75° activation-to-torque ratios were performed for VL, VM, and RF using paired *t*-tests. Because three primary muscle-wise angle comparisons were evaluated, Holm-adjusted *p*-values were also reported as a multiplicity-control sensitivity analysis for the primary angle effect [22]. Bias-corrected and accelerated bootstrap confidence intervals were calculated for mean paired differences [23]. Wilcoxon signed-rank tests were reported as nonparametric sensitivity analyses because of the small sample size. Standardized paired effects were calculated as Hedges-corrected paired standardized mean differences based on the within-participant change scores [24], using the equation g_z = J × d_z, where d_z = mean(D)/SD(D), D is the paired difference score (45° − 75°), and J = 1 − 3/(4df − 1) is the small-sample correction factor with df = *n* − 1. Spearman correlations examined exploratory associations between activation-to-torque ratios and CMJ variables. Because 24 exploratory correlations were tested, Benjamini–Hochberg false-discovery-rate adjustment was applied across the full correlation family [25]. Exploratory simple linear models were used with relative PMax as the dependent variable. Regression results were interpreted using the direction and uncertainty of B coefficients, R^2^, adjusted R^2^, and *p*-values. Statistical significance was set at *p* < 0.05, but because of the exploratory design and small sample size, effect sizes, confidence intervals, multiplicity-adjusted results, and consistency across analyses were prioritized over isolated *p*-values. Summary results were visualized using a study workflow schematic and a forest plot of mean paired differences; a simplified exploratory correlation heatmap is provided as Supplementary Figure S2. A post hoc sensitivity analysis based on Fisher’s z transformation estimated the approximate minimum detectable absolute correlation for *n* = 17, 80% power, and a two-sided α of 0.05. An additional conservative benchmark used α = 0.05/24 to reflect the 24 exploratory correlations. These calculations were used only to describe the study’s sensitivity and not as retrospective evidence of adequate statistical power. Exploratory post hoc analyses also examined whether the within-participant angle difference in each muscle’s activation-to-torque ratio (45° − 75°) varied by sex or was associated with body composition. Sex-group differences were evaluated using exact two-sided permutation tests. Associations with body mass and fat-free mass were assessed using Spearman correlations with two-sided permutation *p*-values based on 20,000 random permutations. These analyses were not prespecified and were interpreted as exploratory. Local subcutaneous adipose tissue thickness was not measured. All statistical analyses were conducted using IBM SPSS Statistics, version 27 (IBM Corp., Armonk, NY, USA).

## 3. Results

### 3.1. Participant Characteristics and CMJ Outcomes

Seventeen participants were included in the analysis. The sample comprised 11 men and 6 women, with a mean age of 20.94 ± 2.08 years. Body mass was obtained from the InBody assessment and used for body-mass-normalized outputs where applicable. Body mass was 75.00 ± 17.32 kg, and fat-free mass was 62.68 ± 16.53 kg. CMJ-derived outcomes, retained from the best valid trial defined by the highest jump height, were: jump height (32.33 ± 7.90 cm), relative PMax (60.08 ± 16.75 W/kg), VMax (3.01 ± 0.47 m/s), FMax (24.21 ± 4.85 N/kg), and CMJ-derived RFD (41.03 ± 33.87 N/kg/s). Participant characteristics and CMJ outcomes are summarized in Table 1.

### 3.2. Angle Differences in Activation-to-Torque Ratios

Activation-to-torque ratios showed consistently higher values at 45° than at 75° for all three quadriceps muscles. For VL, the ratio was 2.59 ± 1.39 at 45° and 1.86 ± 0.76 at 75°, giving a mean paired difference of 0.74 μV/(N·m) (BCa 95% CI: 0.34 to 1.34; paired *t*-test *p* = 0.010; Holm-adjusted *p* = 0.020; Wilcoxon *p* = 0.007; standardized paired effect = 0.68). For VM, the corresponding values were 2.32 ± 1.05 at 45° and 1.79 ± 1.48 at 75°, with a mean paired difference of 0.52 μV/(N·m) (BCa 95% CI: 0.09 to 0.72; paired *t*-test *p* = 0.003; Holm-adjusted *p* = 0.009; Wilcoxon *p* = 0.003; standardized paired effect = 0.83). For RF, the ratio was 3.08 ± 1.63 at 45° and 2.38 ± 0.83 at 75°, with a mean paired difference of 0.69 μV/(N·m) (BCa 95% CI: 0.22 to 1.32; paired *t*-test *p* = 0.028; Holm-adjusted *p* = 0.028; Wilcoxon *p* = 0.035; standardized paired effect = 0.56).

**Figure 2:**
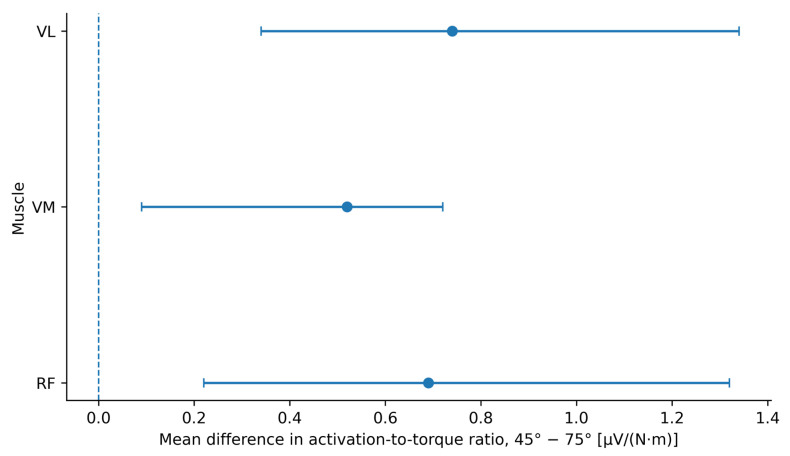
Mean paired angle differences in activation-to-torque ratios. Points indicate mean paired differences calculated as 45° minus 75°, and horizontal bars show BCa 95% confidence intervals. The dashed vertical line indicates no angle difference. Positive values indicate higher activation-to-torque ratios at 45° than at 75°. The x-axis is expressed in μV/(N·m); the muscle axis is categorical. Full *p*-values, Holm-adjusted *p*-values, confidence intervals, and standardized paired effects are reported in Table 2.

All three BCa confidence intervals were entirely above zero, and the inference was unchanged after Holm correction across the three primary muscle-wise angle comparisons. This supports a consistent angle-related pattern in the present sample, while the small sample size means that the precision of the estimates remains limited. Figure 2 was therefore simplified to emphasize the direction and uncertainty of the mean paired differences, while the detailed inferential statistics are retained in Table 2.

### 3.3. Associations with CMJ Performance

The correlation analysis used relative PMax values (W/kg). Across the 24 exploratory Spearman correlations, coefficients ranged from −0.38 to 0.32. No activation-to-torque ratio showed a statistically significant association with jump height, relative PMax, VMax, or CMJ-derived RFD. After Benjamini–Hochberg false-discovery-rate control across the 24 exploratory correlations [25], all adjusted q-values were at least 0.80. The largest negative coefficient was observed between RF ratio at 45° and jump height (ρ = −0.38; *p* = 0.138), and the largest positive coefficient was observed between RF ratio at 45° and CMJ-derived RFD (ρ = 0.32; *p* = 0.213). For relative PMax, the largest absolute coefficient was small to moderate (VL ratio at 45°: ρ = 0.25; *p* = 0.343). These values indicate that the CMJ associations were weak to moderate, statistically uncertain, and unsuitable for performance-prediction claims in this sample. With *n* = 17 and 80% power, the approximate minimum detectable absolute correlation was |r| = 0.63 at a two-sided α of 0.05 and |r| = 0.78 using the conservative α = 0.05/24 benchmark. The observed coefficients were below these approximate detection thresholds. Accordingly, the nonsignificant CMJ findings do not exclude small-to-moderate associations and should be interpreted as inconclusive rather than as evidence of no relationship.

The exact Spearman coefficients and *p*-values are reported in Table 3. A simplified heatmap is provided as Supplementary Figure S2 for visual inspection of the overall exploratory pattern.

### 3.4. Exploratory Regression Analysis

Exploratory simple linear models were recalculated with relative PMax (W/kg) as the dependent variable. Individual ratios and the composite predictor explained very little variance in relative PMax, with R^2^ values ranging from 0.007 to 0.018 and adjusted R^2^ values remaining negative (−0.059 to −0.048). The largest R^2^ was observed for VL ratio at 75° (R^2^ = 0.018), but its regression coefficient was uncertain and the confidence interval crossed zero (B = −2.94 W/kg per unit increase; 95% CI: −14.97 to 9.08; *p* = 0.610). VL ratio at 45° (B = 1.02; 95% CI: −5.58 to 7.62; *p* = 0.746) and RF ratio at 45° (B = −1.07; 95% CI: −6.71 to 4.57; *p* = 0.691) also showed no meaningful explanatory value in this sample.

The composite predictor also did not improve explanatory performance (B = 3.09; 95% CI: −17.12 to 23.30; R^2^ = 0.007; adjusted R^2^ = −0.059; *p* = 0.749). Therefore, the regression analysis is retained as a numerical sensitivity check in Table 4, rather than presented as a main figure.

### 3.5. Exploratory Sex and Body-Composition Analyses

No statistically detectable sex difference was observed in the angle-related ratio change for VL (women − men mean difference = −0.40 μV/(N·m), permutation *p* = 0.488), VM (−0.34 μV/(N·m), *p* = 0.303), or RF (0.77 μV/(N·m), *p* = 0.212). Spearman correlations of the angle-related changes with fat-free mass ranged from −0.25 to 0.11, and correlations with body mass ranged from −0.27 to 0.18; all permutation *p*-values were ≥0.298. Full results are reported in Supplementary Table S1. Because the sample included only six women and eleven men, these estimates were imprecise and should not be interpreted as evidence that sex or body composition has no influence on the ratio.

## 4. Discussion

### 4.1. Principal Findings

This exploratory cross-sectional study examined quadriceps activation-to-torque ratios during maximal isometric knee extension at two knee-flexion angles. The main finding was that activation-to-torque ratios for the vastus lateralis (VL), vastus medialis (VM), and rectus femoris (RF) were higher at 45° than at 75°. In practical terms, this means that, in the present sample, higher surface EMG amplitude per unit torque was observed at the 45° testing angle. The effect was consistent across all three muscles, with positive BCa confidence intervals, agreement between paired *t*-tests and Wilcoxon sensitivity tests, and unchanged inference after Holm correction across the three primary muscle-wise comparisons. The standardized paired effects were moderate in magnitude, but the sample size was small and the estimates should be treated as preliminary.

A second important finding was that the corrected countermovement jump (CMJ) analyses did not support the earlier interpretation that selected ratios strongly predicted jump peak power. After confirming that PMax was expressed as relative peak power in W/kg, the associations between activation-to-torque ratios and CMJ outcomes were weak to moderate, statistically nonsignificant, and not robust after false-discovery-rate adjustment. The strongest conclusion from this study concerns angle-dependent activation demand during isometric knee extension, whereas the CMJ analyses should be interpreted as exploratory and hypothesis-generating.

### 4.2. Interpretation of Angle-Dependent Activation-to-Torque Ratios

The angle-dependent results are biomechanically plausible. Changing knee-flexion angle alters quadriceps muscle length, tendon configuration, joint moment arm, patellofemoral mechanics, and the region of the force–length relationship in which force is produced [1,2,3,4]. Because the activation-to-torque ratio combines an electrical signal with a mechanical output, a higher value can arise from increased EMG amplitude, reduced torque production, or a combination of both. The present finding of higher ratios at 45° is therefore compatible with higher EMG amplitude per unit torque at this joint configuration, although the present design cannot determine whether this reflects neural drive, mechanical disadvantage, force-transmission differences, or measurement variability.

Three broad categories of influence should be distinguished when interpreting this ratio. Neural factors include voluntary drive, motor-unit recruitment and discharge behavior, and muscle-specific activation strategies. Mechanical factors include muscle length, moment-arm geometry, tendon configuration, patellofemoral mechanics, and force transmission. Methodological factors include electrode placement, subcutaneous tissue thickness, signal-processing choices, analysis-window selection, gravity correction, dynamometer positioning, and the use of average peak torque rather than window-matched torque. Because these domains were not independently quantified, the higher ratios observed at 45° cannot be attributed to a single neural or mechanical mechanism.

This interpretation should be kept distinct from the concept of superior neuromuscular efficiency. In some contexts, high EMG amplitude may reflect greater voluntary neural drive, stronger motor-unit recruitment, or an enhanced capacity to activate the muscle. However, when EMG is divided by torque, a higher ratio may also indicate that more activation is needed to produce a given mechanical output. Recent consensus guidance on EMG-to-force estimation and systematic evidence on electromechanical-efficiency indices support this cautious interpretation [9,10]. For this reason, the present study deliberately uses the term activation-to-torque ratio rather than treating the ratio as a direct measure of efficiency. This wording is more conservative and better reflects the descriptive nature of the measurement.

The present results are also compatible with the view that angle-specific testing may provide information that is not captured by a single isometric test angle. If torque and EMG are measured at only one joint position, meaningful variation in activation demand across the operating range of the quadriceps may be missed. Assessing multiple angles can therefore help describe whether a participant produces torque with relatively low or high EMG amplitude per unit torque under different muscle–tendon configurations. Nevertheless, this should be considered a descriptive profiling approach rather than a diagnostic or performance-prediction tool at this stage.

### 4.3. Muscle-Specific Considerations

The similar direction of change across VL, VM, and RF suggests that the angle effect was not restricted to one quadriceps head. However, the physiological meaning of the ratio may differ between muscles. VL and VM are monoarticular knee extensors, whereas RF crosses both the hip and knee joints. Because testing was performed in a seated posture, RF length and activation may have been influenced by the fixed hip position as well as the tested knee angle [3]. As a result, the present design cannot fully separate the influence of knee angle from the broader hip–knee muscle–tendon configuration affecting RF.

This issue is particularly relevant when interpreting RF results. A biarticular muscle may respond differently to changes in joint configuration compared with monoarticular muscles because its functional length depends on two joints. Future studies could improve mechanistic interpretation by systematically manipulating both hip and knee angles, or by combining sEMG with ultrasound-based measures of fascicle length, pennation angle, and tendon behavior [3,9]. Such designs would clarify whether activation-to-torque behavior differs between the vasti and RF because of muscle architecture, joint configuration, or task-specific neural control.

### 4.4. Relationship with Countermovement Jump Performance

The CMJ analyses were included as a broad external exploratory comparison rather than as a validation test or a direct mechanistic analysis of the activation-to-torque ratio. CMJ performance is a dynamic, multi-joint movement that depends on whole-body coordination, lower-limb and trunk contribution, stretch–shortening-cycle mechanics, tendon stiffness, body mass, movement strategy, and the timing of force application [11,12,13,14,15]. By contrast, the activation-to-torque ratio in the present study was obtained during single-joint maximal isometric knee extension. The observed coefficients were small to moderate, none reached conventional significance, and none survived FDR correction. These results therefore provide no evidence of a consistent association in this sample, but the limited statistical sensitivity means that small-to-moderate relationships cannot be excluded.

Accordingly, the CMJ analyses should be interpreted as hypothesis-generating external comparisons, not as evidence that the ratio captures the mechanisms underlying whole-body jump performance. The corrected analysis suggests that the activation-to-torque ratio may be more suitable for describing angle-specific neuromuscular behavior than for explaining jump performance. The study does not show that the ratios are validated markers of explosive performance; it shows that these ratios differ by joint angle and that their relationships with CMJ outcomes remain uncertain.

It is also possible that a stronger relationship would be observed if the mechanical outcome were more closely matched to the test. For example, angle-specific isometric torque or knee-extension impulse may be more directly related to activation-to-torque ratios than whole-body CMJ peak power. Conversely, CMJ variables may be better explained by integrated models that include hip and ankle strength, tendon stiffness, body composition, coordination variables, and movement kinematics in addition to quadriceps EMG and torque. Future work should therefore examine whether activation-to-torque ratios add explanatory value beyond conventional strength and performance measures.

### 4.5. Importance of Unit Consistency and Corrected PMax Interpretation

A key methodological lesson from the present revision is the importance of unit consistency. The initial interpretation suggested stronger associations with PMax, but this was affected by treating peak power values inconsistently. In the corrected analysis, PMax was treated as relative peak power in W/kg, as exported from the OptoJump/GYKO output for the best valid CMJ trial. This correction substantially changed the regression coefficients, apparent effect sizes, and the interpretation of the models. The revised results therefore no longer support a strong performance-prediction claim.

This point is important because absolute power and relative power answer different questions. Absolute power is influenced strongly by body mass and may favor heavier participants, whereas relative power expresses power output per kilogram of body mass and is often more appropriate when comparing individuals of different body sizes. Similarly, standardized or composite scores require careful definition and interpretation before they are used as predictors [26]. Mixing absolute and relative units can create misleading associations. Therefore, all performance variables should be clearly labeled, and future analyses should predefine whether absolute or relative mechanical outputs are being tested.

### 4.6. Composite Index Interpretation

The composite activation-to-torque index should be interpreted with particular caution. Combining several muscle- and angle-specific variables into a single score may be conceptually attractive because neuromuscular function is not determined by one muscle or one joint angle alone. However, post hoc composites can easily overfit small datasets, especially when the same data are used both to select the variables and to test the resulting index. In the corrected analysis, the composite index explained very little variance in relative PMax and was not statistically significant.

For this reason, the composite should not be presented as a validated predictor. At most, it can be described as an exploratory sensitivity analysis that may inform future hypothesis development. A stronger future approach would be to define the composite a priori based on theory, test it in a larger sample, and evaluate its stability using cross-validation or independent replication. Without such validation, the composite index should not be used for applied classification or individual decision-making.

### 4.7. Practical Implications

At present, activation-to-torque ratios should be regarded as exploratory, group-level research variables. The present study did not establish within-session or between-session reliability, typical error, coefficient of variation, or minimal detectable change. Consequently, these ratios should not yet be used for individual profiling, longitudinal monitoring, rehabilitation decisions, or training prescription. Their potential applied value depends on prospective reliability studies demonstrating acceptable measurement stability and sensitivity to meaningful change.

In research settings, angle-specific activation-to-torque ratios may complement conventional group-level measures such as peak torque, jump height, power output, CMJ-derived RFD, and movement kinematics. However, the present findings do not establish that the ratios provide reliable individual-level information or detect meaningful longitudinal change. Any applied interpretation therefore remains preliminary until reliability, responsiveness, and criterion validity have been demonstrated.

### 4.8. Methodological Considerations

Several methodological issues should be considered when interpreting activation-to-torque ratios. Surface EMG is influenced by electrode placement, inter-electrode distance, skin preparation, adipose tissue thickness, crosstalk from adjacent muscles, filtering choices, and the selected analysis window [5,7,8]. The present protocol used standardized skin preparation, anatomical electrode placement, separate Noraxon EMG and Biodex torque acquisition during corresponding MVC test conditions, manual matching by participant and knee angle, and archived Noraxon Standard EMG Analysis output to extract mean amplitude from marked valid MVC periods. Nevertheless, because the ratio depends on two separately recorded signals, errors in either measurement and uncertainty in manual condition matching can influence the final value.

From a sensor-method perspective, the present study is best viewed as an exploratory combination of wireless sEMG and separately acquired dynamometry-derived mechanical output rather than a validation of a synchronized system, new device, or algorithm. Recent work on wearable and portable sport-biomechanics devices and contemporary sEMG development emphasizes that acquisition hardware, integration procedures, processing transparency, and biomechanical context are central to interpreting sensor-derived neuromuscular signals [27,28,29].

The choice of EMG amplitude metric may also affect interpretation. Mean amplitude, MAV, RMS, and integrated EMG can provide related but not identical representations of muscle activation. EMG normalization guidance also emphasizes that the selected amplitude-processing approach should match the intended interpretation and should be reported transparently [6,7]. In this study, the EMG numerator was the Noraxon-exported mean amplitude rather than a separately reconstructed raw-signal RMS or MAV value. Similarly, using separately recorded Biodex average peak torque rather than torque averaged over a time-aligned EMG analysis window may influence the ratio. Future studies should prospectively save raw EMG and torque time series, document exact processing settings and analysis-window definitions, and use a shared hardware trigger or validated software synchronization procedure before evaluating alternative ratio definitions.

The CMJ measurement system also requires careful interpretation. OptoJump has shown strong validity and reliability for estimating vertical jump height [19], and GYKO has been examined for concurrent validity in vertical jump assessment [21]. However, temporal and derivative variables can be influenced by system settings, sensor placement, movement execution, and trial-selection rules [20]. Therefore, CMJ-derived variables should be interpreted in relation to the specific equipment, software settings, and testing protocol used.

Exploratory analyses did not identify statistically detectable sex differences in the angle-related ratio changes or associations with body mass or fat-free mass. However, the small and imbalanced sample provides limited precision, and local subcutaneous adipose tissue thickness—an important determinant of surface EMG amplitude—was not measured. These findings therefore cannot exclude sex- or body-composition-related effects.

The activation-to-torque ratio combined Noraxon-exported mean EMG amplitude with Biodex average peak torque obtained from a separately recorded, manually matched test condition. Because the systems did not share an electronic trigger or common time base, the electrical and mechanical variables were not sample-level-synchronized or derived from identical time-aligned analysis windows. A window-matched approach would provide greater temporal consistency, but retrospective reconstruction was not possible because only Biodex summary variables (e.g., peak torque, average peak torque, normalized torque, and impulse) were preserved and no time-aligned Biodex torque series corresponding to the manually selected EMG periods was available. Therefore, the predefined condition-matched ratio was retained as the primary outcome. Future studies should use direct synchronization and calculate both EMG amplitude and torque over identical time-aligned windows to determine whether the observed angle-dependent differences are robust to the acquisition and denominator definitions.

Dynamometer-based lower-extremity strength testing can provide valid and reliable outputs when protocols are standardized, but absolute values remain dependent on device setup, positioning, normalization, and data-processing choices [30].

### 4.9. Limitations

This study has several limitations. First, the sample size was small (*n* = 17), which limits statistical power and increases uncertainty around correlations and regression estimates. Small samples are also more sensitive to individual variability and influential observations. Second, the cross-sectional design prevents causal inference. The present data cannot determine whether changing activation-to-torque ratios would lead to changes in torque or jump performance. Third, EMG summary values were obtained from archived Noraxon Standard EMG Analysis exports. The available records confirmed the acquisition frequency, marked analysis periods, and mean-amplitude summaries, but they did not fully preserve all internal preprocessing parameters, such as exact filter type/order, rectification procedure, and amplitude-processing algorithm. The EMG numerator should therefore be interpreted as the exported Noraxon mean-amplitude metric, not as a fully reconstructable raw-signal feature. The sensitivity analysis indicated that the study was not sufficiently powered to reliably detect small-to-moderate correlations; therefore, nonsignificant CMJ associations should be considered inconclusive rather than evidence of no relationship.

Fourth, gravity correction was not applied in the Biodex export. Because limb-weight contribution can vary across joint positions, angle-specific gravitational effects may have influenced absolute torque values and, consequently, the activation-to-torque ratios. This issue is relevant because gravity-correction procedures can affect dynamometer torque interpretation [31]. The angle-related findings should therefore be interpreted as protocol-specific EMG-amplitude-to-Biodex-output torque differences rather than fully gravity-corrected estimates of quadriceps mechanical output.

Fifth, the EMG numerator and torque denominator were acquired using separate systems and were manually matched by participant, knee angle, and test set rather than electronically synchronized. No shared time base or time-aligned Biodex torque series was preserved; consequently, a retrospective window-matched sensitivity analysis could not be performed. Future studies should prospectively record EMG and torque using a validated synchronization method, calculate both variables over identical time-aligned analysis windows, and compare these findings with conventional peak-torque-based ratios.

Sixth, the participants were young adults but were not clearly defined as competitive athletes. Therefore, the findings should not be generalized to elite sport populations without further validation. Seventh, the study did not include direct measures of muscle architecture, tendon stiffness, electromechanical delay, or motor-unit behavior. These measures would help explain whether the observed angle-dependent ratios were primarily related to neural drive, muscle–tendon mechanics, or force-transmission properties. Eighth, reliability was not directly tested in the present dataset. Future work should include test–retest reliability, typical error, coefficient of variation, and minimal detectable change for each activation-to-torque ratio. Ninth, the Biodex protocol was brief because of testing-time constraints. It used two 3 s isometric knee-extension contractions per angle, with a 10 s relaxation period and standardized rapid-and-hard verbal instructions. Therefore, replication studies should examine whether the same angle-dependent pattern is observed under longer contraction durations, more repeated trials, and separate test–retest sessions.

### 4.10. Future Directions

Future studies should build on these preliminary findings using larger samples, predefined hypotheses, and reliability-focused designs. A useful next step would be to test whether activation-to-torque ratios are stable across repeated sessions and whether they change after targeted training. Longitudinal studies could determine whether short- or long-muscle-length training modifies angle-specific ratios and whether those changes correspond to improvements in torque, tendon stiffness, or sport-specific performance [4,11,12,13,14,15].

Future research should also examine whether activation-to-torque ratios provide incremental information beyond conventional mechanical measures. This could be tested using multivariable models that include peak torque, body composition, jump mechanics, CMJ-derived performance variables, and EMG-derived variables. Because EMG-based force estimation is limited without a mechanical and physiological context, these models should be interpreted cautiously and ideally validated in independent samples [9,10]. If the ratio explains unique variance beyond simpler measures, its applied relevance would be strengthened. If not, it may remain most useful as a descriptive research variable rather than a monitoring tool.

## 5. Conclusions

In conclusion, quadriceps activation-to-torque ratios showed consistent angle-dependent differences during maximal isometric knee extension, with higher ratios at 45° than at 75° for VL, VM, and RF. This pattern is compatible with higher Noraxon-exported EMG amplitude per unit Biodex-reported torque at the 45° testing angle in the present sample, although the underlying neural and mechanical contributors cannot be separated from the present design, and angle-dependent gravitational contributions to the Biodex-reported torque values cannot be excluded. Corrected analyses using relative CMJ peak power did not support strong associations with jump performance. Therefore, the findings should be interpreted as preliminary evidence for angle-specific EMG amplitude per unit torque, not as validated evidence for performance prediction. Larger studies with predefined predictors, reliability testing, gravity-corrected torque outputs, prospectively specified EMG processing, and longitudinal designs are needed before activation-to-torque ratios can be recommended for applied monitoring or decision-making.

## Figures and Tables

**Table 1:** Participant characteristics and CMJ outcomes. Values are mean ± SD unless otherwise indicated. Body mass was obtained from the InBody assessment and used for body-mass-normalized outputs where applicable. PMax = relative peak power; VMax = maximum velocity; FMax = maximum force; RFD = CMJ-derived rate of force development (N/kg/s). CMJ outcomes represent the best valid hands-on-hips CMJ trial, defined by the highest jump height among three maximal trials.

Variable	Mean ± SD/*n*
*N*	17
Age (years)	20.94 ± 2.08
Sex	11 men, 6 women
Body mass (kg; InBody)	75.00 ± 17.32
Fat-free mass (kg)	62.68 ± 16.53
Jump height (cm)	32.33 ± 7.90
PMax (W/kg)	60.08 ± 16.75
VMax (m/s)	3.01 ± 0.47
FMax (N/kg)	24.21 ± 4.85
CMJ-derived RFD (N/kg/s)	41.03 ± 33.87

**Table 2:** Comparison of activation-to-torque ratios between 45° and 75° knee flexion. Ratios were calculated as Noraxon-exported mean EMG amplitude divided by average peak torque (μV/(N·m)). BCa = bias-corrected and accelerated bootstrap confidence interval; W *p* = Wilcoxon signed-rank *p*-value; Holm *p* = Holm-adjusted *p*-value for the three primary muscle-wise angle comparisons. Std. effect = Hedges-corrected paired standardized mean difference based on within-participant change scores, calculated as J × [mean(D)/SD(D)], where D = 45° − 75° and J is the small-sample correction factor.

Muscle	45° Ratio Mean ± SD	75° Ratio Mean ± SD	Δ 45° − 75°	BCa 95% CI	t *p*	W *p*	Std. Effect	Holm *p*
VL	2.59 ± 1.39	1.86 ± 0.76	0.74	0.34, 1.34	0.010	0.007	0.68	0.020
VM	2.32 ± 1.05	1.79 ± 1.48	0.52	0.09, 0.72	0.003	0.003	0.83	0.009
RF	3.08 ± 1.63	2.38 ± 0.83	0.69	0.22, 1.32	0.028	0.035	0.56	0.028

**Table 3:** Spearman correlations between activation-to-torque ratios and CMJ variables. No association reached *p* < 0.05, and none survived false-discovery-rate correction. PMax is reported in W/kg. RFD refers to CMJ-derived rate of force development (N/kg/s).

Ratio	Jump Height ρ (*p*)	Pmax ρ (*p*)	Vmax ρ (*p*)	RFD ρ (*p*)
VL ratio (45°)	0.05 (0.837)	0.25 (0.343)	0.12 (0.646)	0.22 (0.400)
VM ratio (45°)	−0.35 (0.163)	−0.27 (0.295)	−0.10 (0.701)	0.23 (0.384)
RF ratio (45°)	−0.38 (0.138)	−0.08 (0.758)	−0.19 (0.474)	0.32 (0.213)
VL ratio (75°)	−0.29 (0.257)	−0.16 (0.529)	0.03 (0.911)	0.26 (0.319)
VM ratio (75°)	−0.33 (0.194)	−0.25 (0.323)	0.06 (0.823)	0.16 (0.548)
RF ratio (75°)	−0.34 (0.185)	−0.10 (0.701)	−0.01 (0.970)	0.03 (0.918)

**Table 4:** Exploratory linear models with relative PMax (W/kg) as the dependent variable. B represents the unstandardized change in relative PMax (W/kg) per one-unit increase in the predictor. All models explained less than 2% of the variance, and all 95% confidence intervals for B crossed zero. These models are retained as exploratory sensitivity analyses and should not be interpreted as validated prediction models.

Predictor	Intercept	B	95% CI for B	R^2^	Adjusted R^2^	*p*
VL_ratio_45	57.43	1.02	−5.58, 7.62	0.007	−0.059	0.746
RF_ratio_45	63.37	−1.07	−6.71, 4.57	0.011	−0.055	0.691
VL_ratio_75	65.54	−2.94	−14.97, 9.08	0.018	−0.048	0.610
Composite	60.08	3.09	−17.12, 23.30	0.007	−0.059	0.749

## Data Availability

The datasets generated and/or analyzed during the current study are available from the corresponding author upon reasonable request.

## Supplementary Materials

The following supporting information can be downloaded at: https://www.mdpi.com/article/10.3390/s26144568/s1, Figure S1: Archived MyoResearch XP EMG acquisition and interface-configuration screens; Figure S2: Exploratory heatmap of activation-to-torque ratio associations with CMJ outcomes; Table S1: Exploratory sex differences and body-composition associations for angle-related changes in quadriceps activation-to-torque ratios; Table S2: Summary of the archived EMG acquisition, manual data-matching, and signal-processing workflow.
