# Supplementary material for: Sensor-Based Assessment of Quadriceps EMG-Amplitude-to-Torque Ratios at Different Knee Angles: An Exploratory Cross-Sectional Study
Source: Sensors (Basel). 2026 Jul 18;26(14):4568. doi: 10.3390/s26144568 (PMC13416507; doi:10.3390/s26144568)
Supplement: Supplementary file 1 [file sensors-26-04568-s001.zip › Supplementary_Figure_S1.pdf]

Supplementary Figure S1. Archived MyoResearch XP EMG acquisition and interface-configuration screens

These screenshots document preserved EMG acquisition and software-configuration information. Participant identifiers have been redacted. The configured Biodex interface shown in Panel B did not yield valid recorded Biodex torque, velocity, or angle outcomes because of a technical connection problem and should not be interpreted as evidence of integrated or electronically synchronized acquisition. The screenshots also do not establish the exact signal-processing filter type or order, rectification procedure, or amplitude-processing algorithm used to generate the exported mean-amplitude summary.

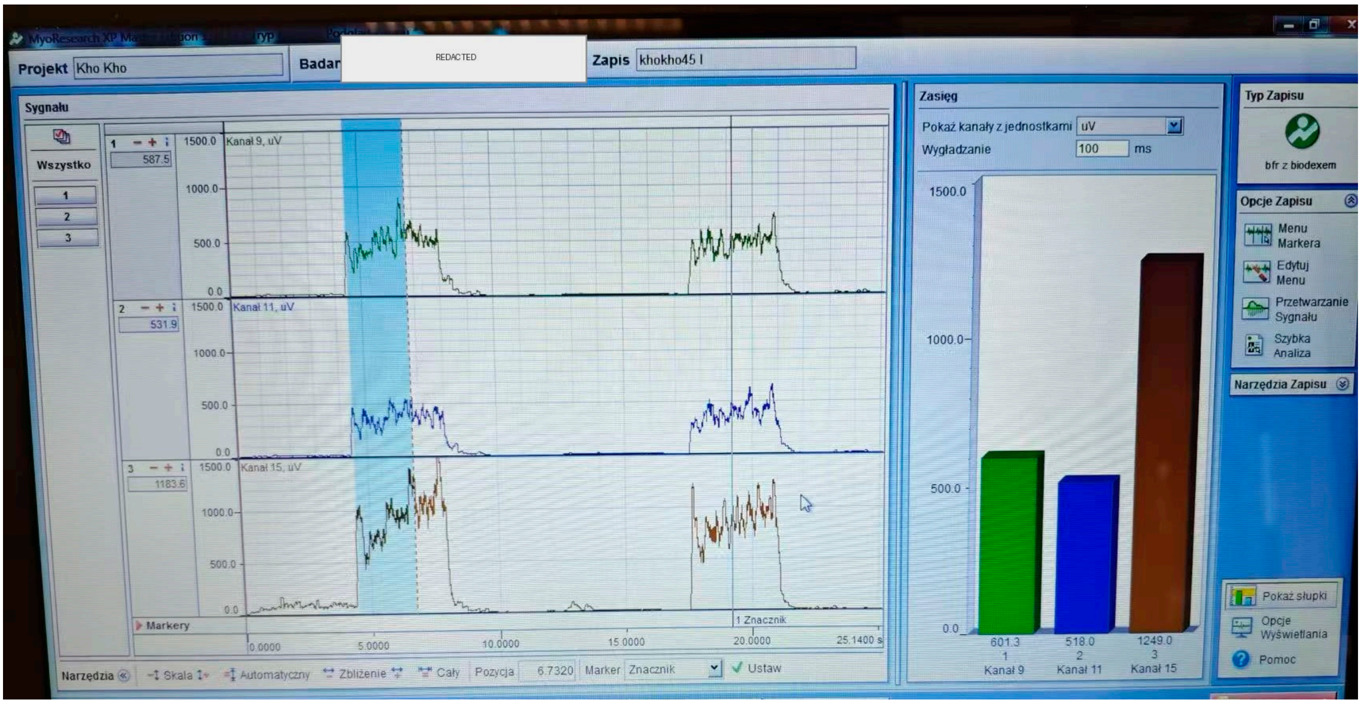

Panel A. MyoResearch XP Master Edition trace view showing three EMG channels (channels 9, 11, and 15) displayed in µV and a manually marked valid MVC analysis period. The visible 100-ms smoothing control is treated as a display/feedback setting and not as evidence of the preprocessing filter applied to the exported amplitude.

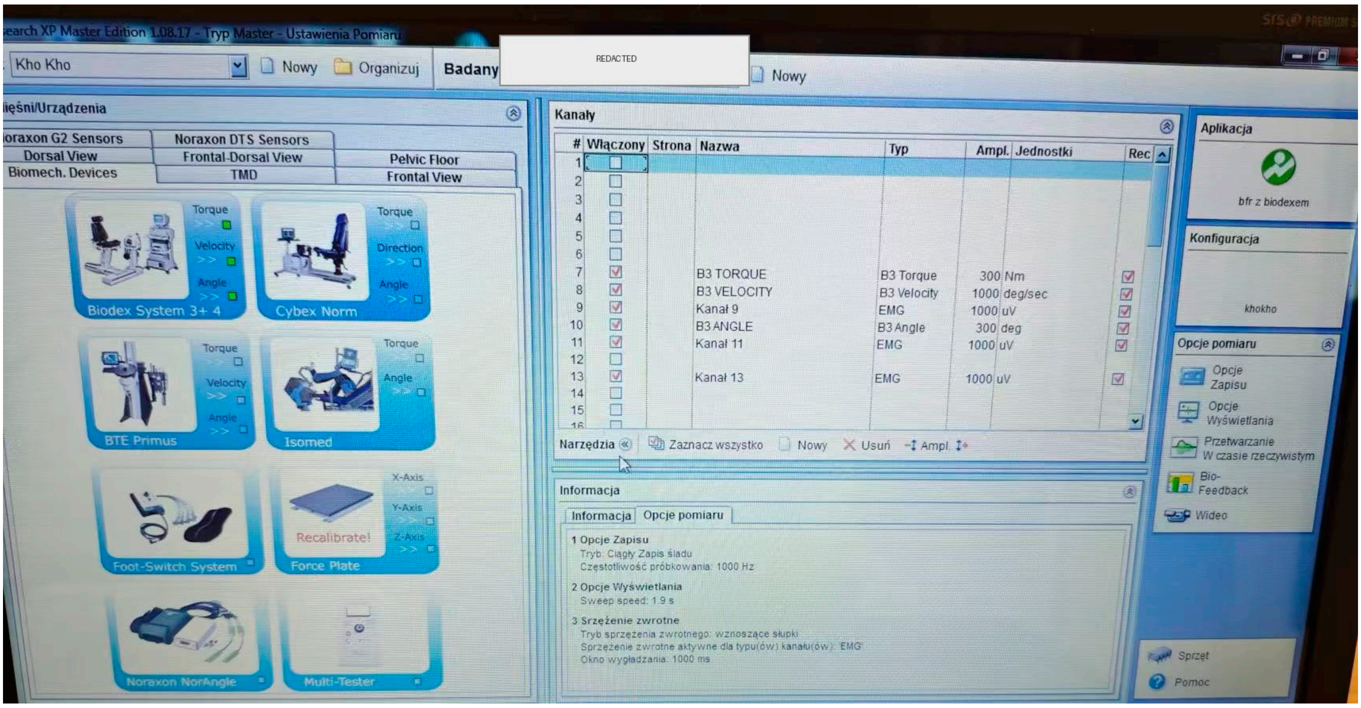

Panel B. MyoResearch XP measurement-configuration screen showing a configured Biodex System 3/4 interface, torque, angular-velocity, angle, and EMG channel ranges, and the archived 1000-Hz Noraxon sampling setting. Because of a technical connection problem, the configured Biodex channels did not provide valid recorded torque, velocity, or angle outcomes in MyoResearch XP. Biodex torque was recorded separately and manually matched to the corresponding participant, knee angle, and test set. The third EMG channel is labelled 13 in this configuration view and 15 in Panel A. The visible 1000-ms smoothing value is treated as a feedback/display setting because its role in the exported EMG amplitude is not documented.
