# Supplementary material for: Sensor-Based Assessment of Quadriceps EMG-Amplitude-to-Torque Ratios at Different Knee Angles: An Exploratory Cross-Sectional Study
Source: Sensors (Basel). 2026 Jul 18;26(14):4568. doi: 10.3390/s26144568 (PMC13416507; doi:10.3390/s26144568)
Supplement: Supplementary file 1 [file sensors-26-04568-s001.zip › Supplementary_Figure_S2.pdf]

Supplementary Figure S2. Exploratory heatmap of activation-to-torque ratio associations with CMJ outcomes

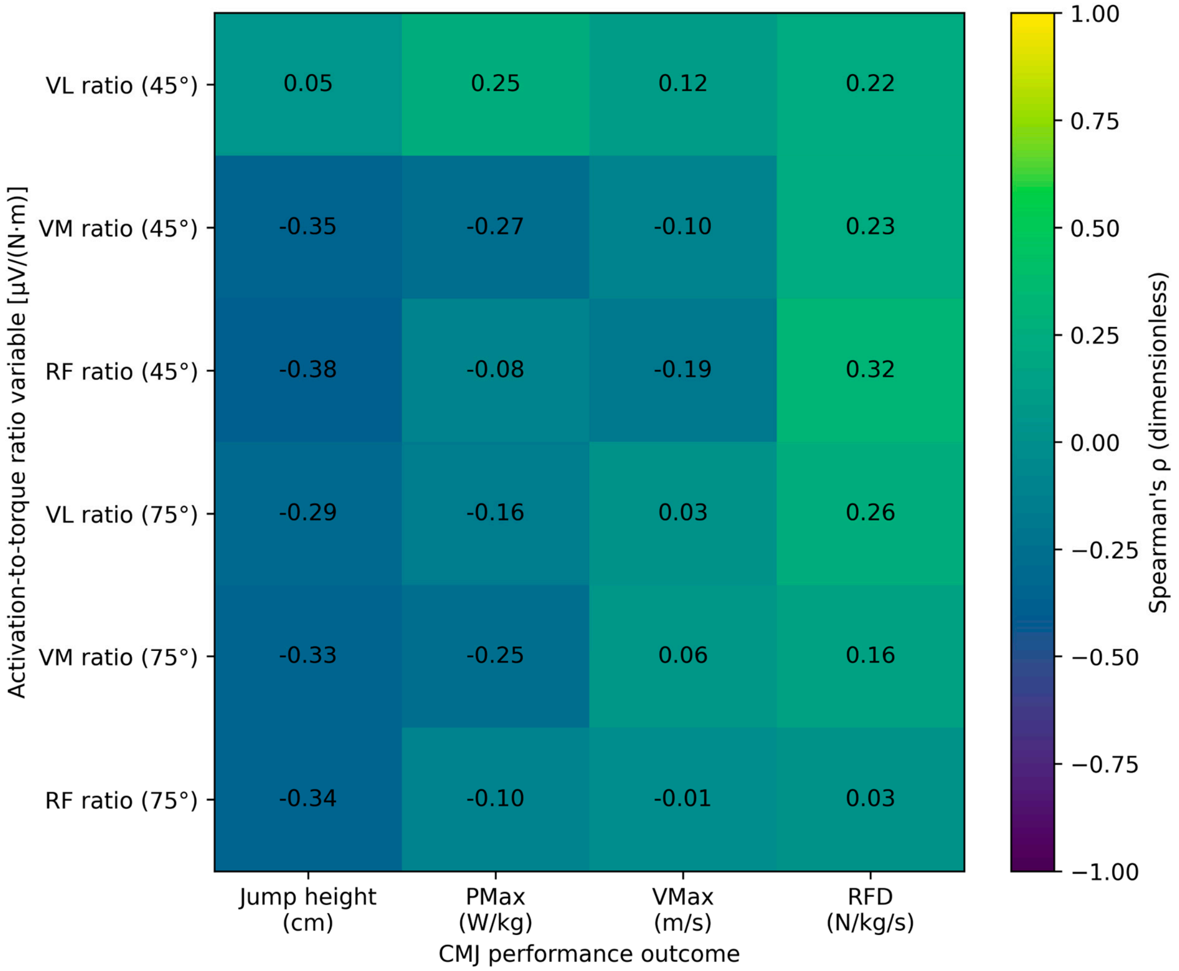

Spearman correlation coefficients between activation-to-torque ratios and CMJ outcomes. CMJ units are shown in the x-axis labels; activation-to-torque ratio variables are expressed in μV/(N·m), and Spearman's ρ is dimensionless. The display is descriptive: no coefficient reached $p < 0.05$, and none survived false-discovery-rate correction. Exact coefficients and p-values are reported in Table 3.
