# Supplementary material for: Sensor-Based Assessment of Quadriceps EMG-Amplitude-to-Torque Ratios at Different Knee Angles: An Exploratory Cross-Sectional Study
Source: Sensors (Basel). 2026 Jul 18;26(14):4568. doi: 10.3390/s26144568 (PMC13416507; doi:10.3390/s26144568)
Supplement: Supplementary file 1 [file sensors-26-04568-s001.zip › Supplementary_Table_S1.pdf]

Supplementary Table S1. Exploratory sex differences and body-composition associations for angle-related changes in quadriceps activation-to-torque ratios

Δ ratio represents the difference between activation-to-torque ratios measured at 45° and 75° knee flexion (45° − 75°), expressed in μV/(N·m). Sex comparisons represent women minus men and were evaluated using exact two-sided permutation tests. Associations with fat-free mass (FFM) and body mass were assessed using Spearman's rank correlation with two-sided permutation p-values based on 20,000 random permutations. All analyses were post hoc and exploratory.

Muscle	Women Δ ratio Mean ± SD	Men Δ ratio Mean ± SD	Women − Men Mean difference (p)	ρ with FFM (p)	ρ with body mass (p)
VL	0.48 ± 1.17	0.88 ± 0.98	−0.40 (0.488)	0.11 (0.667)	0.00 (0.990)
VM	0.30 ± 0.96	0.65 ± 0.28	−0.34 (0.303)	0.11 (0.682)	0.18 (0.493)
RF	1.19 ± 1.58	0.42 ± 0.87	0.77 (0.212)	−0.25 (0.326)	−0.27 (0.298)

FFM = fat-free mass. Local subcutaneous adipose tissue thickness, an important determinant of surface EMG amplitude, was not measured. Therefore, these exploratory analyses should not be interpreted as evidence that sex or body composition has no influence on the activation-to-torque ratio.
