# Supplementary material for: Sensor-Based Assessment of Quadriceps EMG-Amplitude-to-Torque Ratios at Different Knee Angles: An Exploratory Cross-Sectional Study
Source: Sensors (Basel). 2026 Jul 18;26(14):4568. doi: 10.3390/s26144568 (PMC13416507; doi:10.3390/s26144568)
Supplement: Supplementary file 1 [file sensors-26-04568-s001.zip › Supplementary_Table_S2.pdf]

Supplementary Table S2. Summary of the archived EMG acquisition, manual data-matching, and signal-processing workflow

The table distinguishes information preserved in the study archive from parameters and temporal relationships that could not be reconstructed retrospectively. The Biodex and Noraxon systems were operated separately and manually matched at the test-condition level; they were not electronically synchronized.

Workflow stage	Preserved / available information	Unavailable / uncertain information
Acquisition systems and software	Noraxon wireless EMG operated through MyoResearch XP Master Edition; Biodex System 4 Pro used separately for torque measurement	A Biodex System 3/4 interface was configured in MyoResearch XP, but valid Biodex torque, velocity, and angle outcomes were not recorded in the Noraxon files
EMG sampling frequency	1000 Hz in the archived Noraxon acquisition setting	Sampling of Biodex signals within Noraxon was not applicable because valid Biodex signals were not recorded there
Recorded measurements	VL, VM, and RF EMG recorded in Noraxon; torque outcomes recorded separately by Biodex	No valid Biodex torque, angle, or angular-velocity outcome series in the Noraxon archive
Manual coordination and data matching	Records manually matched by participant, knee angle, and corresponding test set	No hardware/software synchronization, shared trigger, common time base, or sample-level temporal alignment
EMG analysis-period selection	Manually marked valid MVC periods visible in archived Noraxon records	Complete time-window definitions for every participant and exact temporal correspondence with Biodex torque
Exported EMG variable	'Averaged Mean Amplitude of All Periods' (μV)	Exact internal amplitude algorithm (e.g., RMS, MAV, or other)
Signal conditioning	Display/feedback smoothing controls visible in screenshots	Exact preprocessing filter type, cut-off frequencies, filter order, and whether the visible smoothing settings affected the exported amplitude
Rectification	Not documented	Rectification procedure
Mechanical denominator	Biodex average peak torque from the separately recorded valid MVC trials in the corresponding manually matched test condition	Mean torque derived over a time-aligned EMG analysis window
Ratio calculation	Noraxon-exported mean EMG amplitude divided by Biodex average peak torque for the corresponding manually matched participant/angle condition	Electronically synchronized or window-matched EMG-to-mean-torque sensitivity ratio

Abbreviations: EMG, electromyography; MAV, mean absolute value; MVC, maximal voluntary contraction; RF, rectus femoris; RMS, root mean square; VL, vastus lateralis; VM, vastus medialis.
